# Case Report: Persistent fetal vasculature associated with lenticular coloboma

**DOI:** 10.3389/fmed.2026.1863134

**Published:** 2026-07-10

**Authors:** Naiyu Sun, Jinchang Tian, Hong Zhang

**Affiliations:** Ophthalmology department, The First Affiliated Hospital of Harbin Medical University, Harbin, China

**Keywords:** AS-OCT, capsular tension ring implantation, case report, lenticular coloboma, persistent fetal vasculature

## Abstract

**Background:**

Persistent fetal vasculature (PFV) is a rare congenital ocular anomaly, featuring typical fibrovascular stalks in the vitreous cavity. PFV may cause anterior and posterior segment anomalies such as leukocoria, microphthalmos, cataract, elongated ciliary processes, optic nerve hypoplasia, intralenticular hemorrhage, retinal dysplasia and strabismus. However, cases of PFV concurrent with lenticular coloboma are rarely reported. This report presents a rare case of anterior PFV associated with lenticular coloboma.

**Case presentation:**

A 23-year-old female presented with poor vision since childhood and deteriorated visual acuity in the right eye for 10 years. Dilated examination showed a localized central posterior subcapsular opacity in the lens, a fibrous stalk on the central posterior capsule extending to the vitreous cavity, elongated ciliary processes on the nasal area. Also a crescent-shaped lens defect and zonular defect was observed at the nasal equatorial region. Anterior segment optical coherence tomography (AS-OCT) examination provided a clear visualization of a band-shaped high reflectivity signal on the central posterior capsule, extending into the vitreous cavity. Lens coloboma along with zonular defect was also visualized by 3D reconstructed images of AS-OCT scan. The patient received an optimized surgery of cataract and anterior vitrectomy, during which a capsular tension ring implantation was made and an IOL was implanted into capsular bag in one surgery. Ethical approval for this observational retrospective study was waived by the Ethics Committee of our hospital and a written informed consent was obtained from the patient for publication of this case report and any accompanying images.

**Conclusion:**

This case represents a rare coexistence of PFV and lens coloboma. The report highlights the distinctive diagnostic value of AS-OCT in identifying anterior PFV and discusses the application of optimized surgical strategies in the management of this complex presentation.

## Introduction

Persistent fetal vasculature (PFV), or persistent hyperplastic primary vitreous (PHPV), is a rare congenital ocular anomaly due to continuous proliferation of the original vitreous and hyaloid vasculature, which failed to regress normally during the embryonic period (1). The classical manifestation of PFV is the remnants of the fetal hyaloid system, which present in different severity, extending from the optic disc to the lens to varying degrees (2). PFV is classified as either an anterior form, primarily affecting the lens, ciliary processes and anterior chamber, or a posterior form, for which retinal folds are the key manifestation. Combined PFV (both anterior and posterior) is the most common and complex form, accounting for about 60% of all cases (3, 4). Associated anterior and posterior segment anomalies may include leukocoria, microphthalmia, cataract, elongated ciliary processes, optic nerve hypoplasia, intralenticular hemorrhage, retinal dysplasia and strabismus (5). Serious potential complications comprise angle-closure glaucoma, hyphema, vitreous hemorrhage, and tractional retinal detachment (6).

The diagnosis of PFV relies on the direct observation of persistent fetal vascular structures. When the refractive media are obscured, B-scan ultrasonography emerges as the most common definitive diagnostic modality, though CT, MRI, and fluorescein angiography are also reasonable options. B-scan is utilized to identify a fibrovascular stalk, intraocular masses, microphthalmia, and calcification. The adjunctive use of Doppler ultrasound is particularly valuable for confirming the vascular nature of the persistent tissue (7).

Surgery is the main treatment of PFV. Anterior or posterior vitrectomy combined with lens extraction can clear the obstructed visual axis, removal of the hyaloid stalk releases retinal and ciliary body traction, reducing the chances of ocular development restriction. Endodiathermy was often used to control intraocular bleeding. Lens extraction with or without IOL implantation may deepens the anterior chamber, which lower the risk of secondary glaucoma (8).

In this article, we describe a case of young adult who came to the clinic with unilateral congenital cataract. Examination through an undilated pupil revealed only a posterior subcapsular opacity and B scan showed no fibrovascular stalk. However, an anterior PFV and lenticular coloboma was detected after dilated slit lamp exam and anterior segment optical coherence tomography (AS-OCT) exam. The patient received anterior vitrectomy and phacoemulsification. A capsular tension ring was placed and an intraocular lens was inserted into the capsular bag.

## Case description

### Patient information

A 23-year-old female presented with poor vision since childhood and deteriorated visual acuity in the right eye for 10 years, the symptom was causeless, painless and progressive. She had myopia and amblyopia in her right eye since childhood. There was no history of ocular trauma, local or systemic steroid use, ocular radiation exposure or other ocular diseases. The patient was healthy and was born full-term via a uncomplicated spontaneous vaginal delivery with no history of infection or radiation exposure during pregnancy. No family history or genetic disorders was found in her family.

### Clinical examination

**Figure 1:**
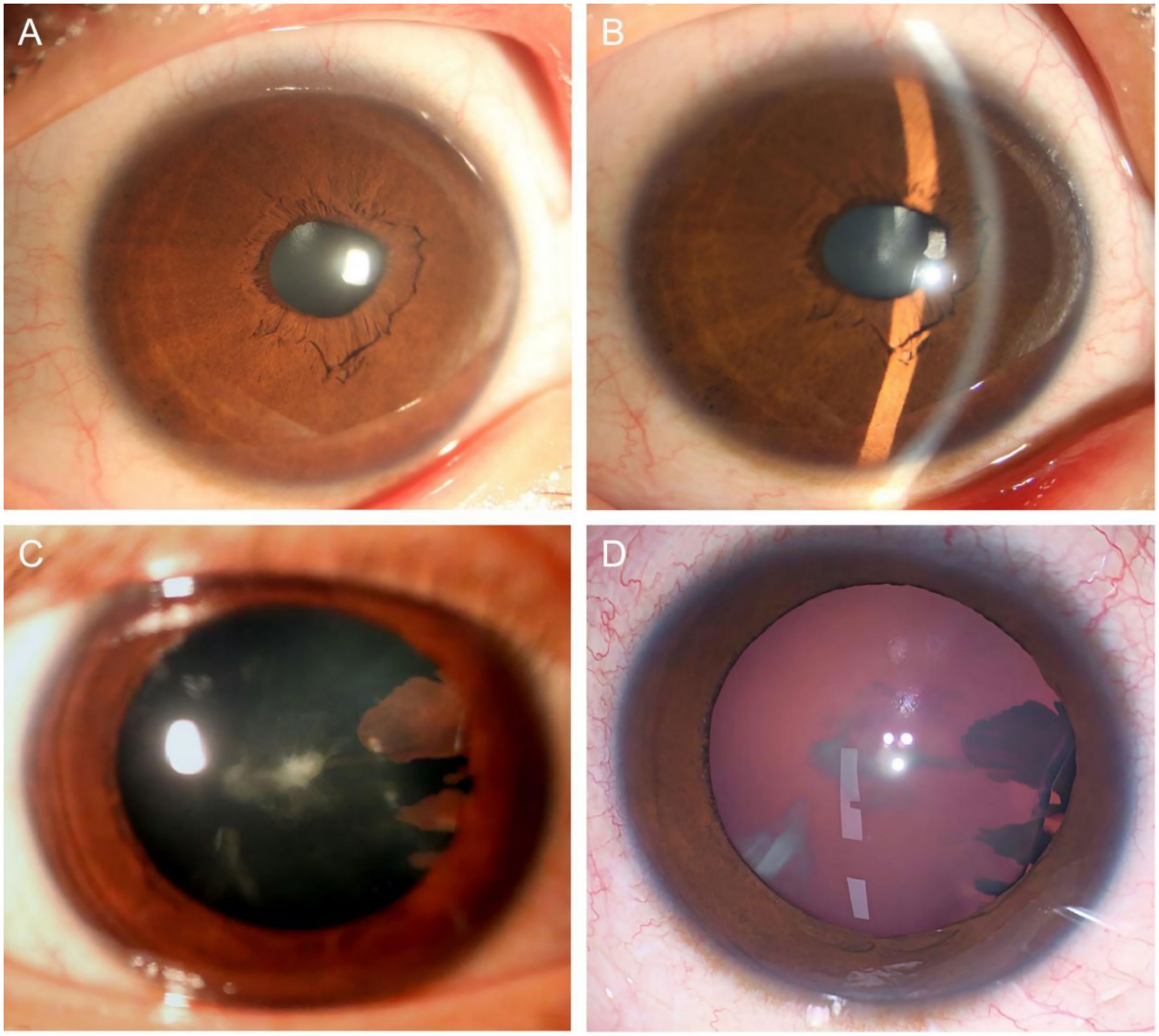
Anterior segment images. **(A)** Anterior segment image under undilated pupil showed irregular pupil with mild nasal displacement; **(B)** undilated exam showed localized central posterior subcapsular opacity in the lens; **(C)** anterior segment image under dilated pupil revealed subcapsular fibrous stalk and elongated ciliary processes; **(D)** regional lens defect and zonular defect, subcapsular fibrous stalk, elongated ciliary processes (under microscope).

The patient’s bilateral best corrected visual acuity (BCVA) was 0.01(OD)/1.0(OS). No abnormalities found in the left eye. In her right eye, an undilated examination showed clear cornea, normal anterior chamber depth, irregular pupil with mild nasal displacement, photoreaction (+), localized central posterior subcapsular opacity in the lens (Figures 1A,B).

**Figure 2:**
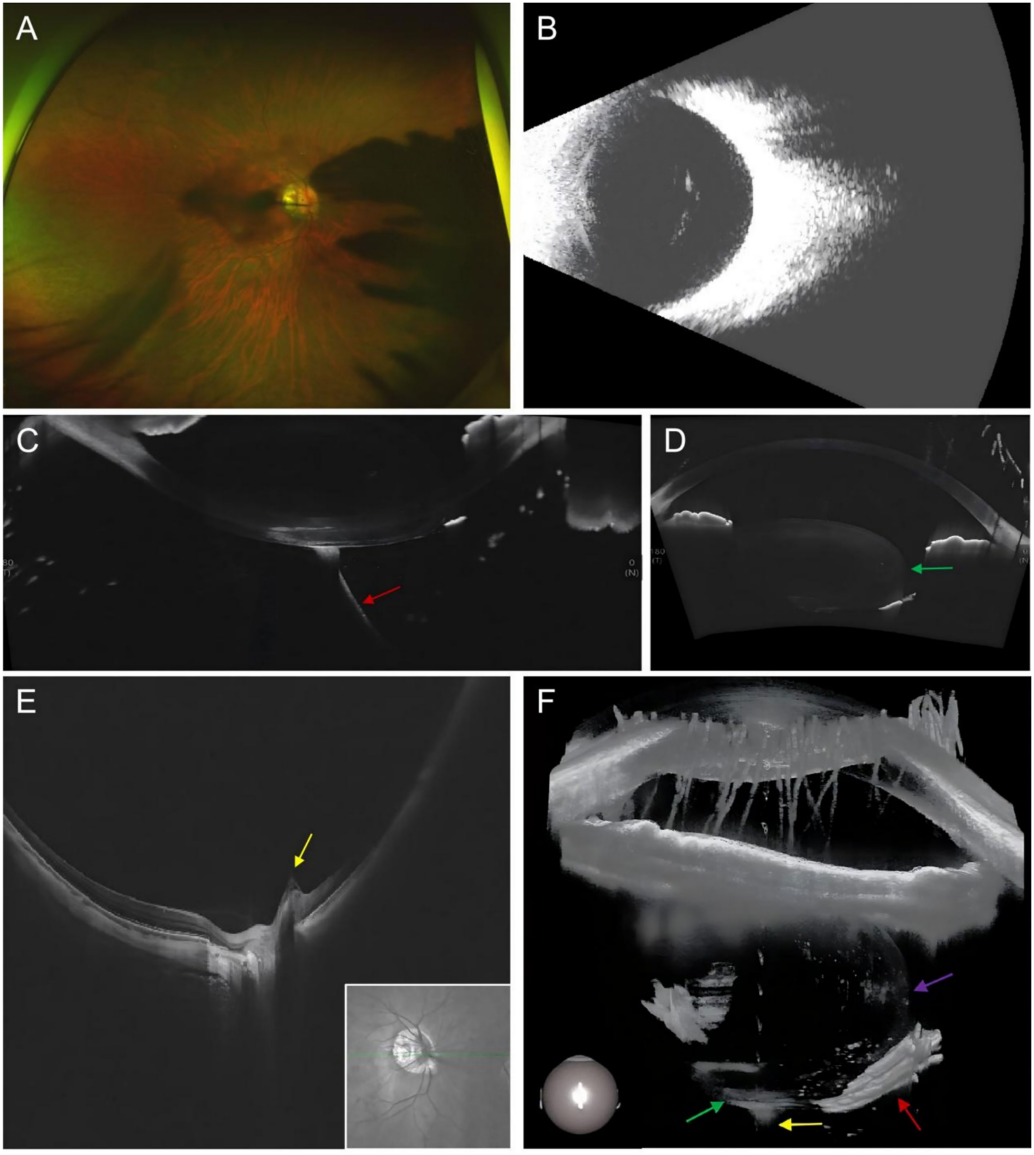
Preoperative ocular examinations. **(A)** Preoperative SLO examination revealed posterior pole obscuration, peripheral opacities, tigroid-patterned appearance and peripapillary atrophy; **(B)** preoperative B-scan ultrasonography showed elongated axial length and vitreous floaters; **(C)** AS-OCT showed a band-shaped high reflectivity signal on the central posterior capsule, extending into the vitreous cavity (red arrow); **(D)** AS-OCT showed regional lens defect and zonular defect on nasal equatorial area (green arrow); **(E)** optic disc OCT revealed a localized short linear medium-intensity signal in the optic disc region (yellow arrow); **(F)** three-dimensional AS-OCT showed pigmented membrane tissue attached to the nasal side of the posterior capsule (red arrow), opacification at the posterior pole of the lens (green arrow), residual primary vitreous adherent posterior to the posterior capsule (yellow arrow), and regional lens defect and zonular defect on on nasal equatorial area (purple arrow).

However, under dilated examination, a fibrous stalk was detected on the central posterior capsule, along with irregularly shaped pigmentation (elongated ciliary processes) on the nasal posterior capsule (Figure 1C). Also, a crescent-shaped lens defect and zonular defect was observed at the nasal peripheral area (Figure 1D). Fundus examination was partially hindered by obscured refractive media. Posterior pole obscuration was observed, along with peripheral opacities consistent with the morphology of elongated ciliary processes. In the visible areas, tigroid-patterned appearance and peripapillary atrophy were noted (Figure 2A).

Intraocular pressure (IOP) was 16 mmHg on both eyes. Standard B-scan Ultrasound only present elongated axial length and mild vitreous floaters in the right eye (Figure 2B). The manifest refraction were −10.75 −1.50 × 172° (OD) and −1.00 −1.00 × 2° (OS). Her preoperative axial length was 27.77 mm (OD) and 23.77 mm (OS), the keratometry values were 45.77 @ 172° and 47.28 @ 82° (1.51 D cyl) in the right eye and 44.62 @ 2° and 45.68 @ 92° (1.06D cyl) in the left eye. ACD were 3.25 mm (OD) and 3.67 mm (OS), LT 4.29 mm (OD) and 3.88 mm (OS). All the above perameters were measured by IOL Master 700.

AS-OCT examination revealed high reflectivity signals in the posterior cortex of the lens. A band-shaped high reflectivity signal was observed on the central posterior capsule, extending into the vitreous cavity (Figure 2C). There was crescent-shaped coloboma on nasal equatorial region of the lens, along with zonular defect on the corresponding area. High reflectivity signals were also detected behind the nasal posterior capsule (Figure 2D). OCT of the macular area showed unclear visualization, while a localized short linear medium-intensity signal was observed in the optic disc region (Figure 2E). Three-dimensional reconstruction provides a more visualized image of the anterior segment OCT (Figure 2F).

### Diagnosis

The final diagnosis was anterior PFV, lenticular coloboma, congenital cataract, pathological myopia and amblyopia in the right eye. A surgical treatment was performed.

### Surgical procedure

**Figure 3:**
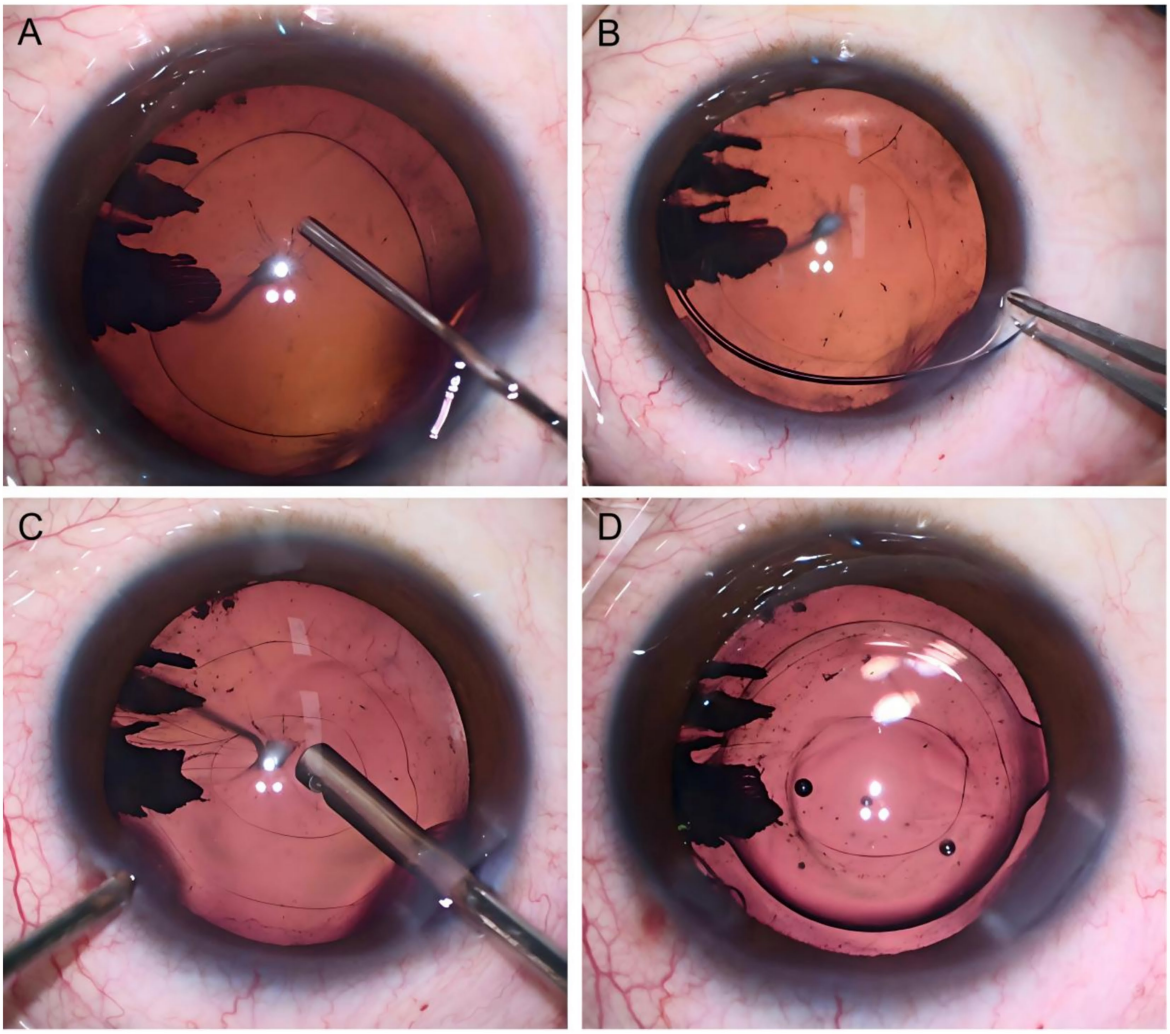
Intraoperative images. **(A)** Non-vascular fibrous stalk extending from the posterior lens capsule into the vitreous cavity with good mobility; **(B)** capsular tension ring implantation; **(C)** 4.0-mm posterior capsulotomy, fibrous tissue excision and anterior vitrectomy; **(D)** a posterior chamber monofocal IOL was implanted into capsular bag.

A surgical treatment of phacoemulsification, anterior vitrectomy, capsular tension ring implantation and IOL implantation was performed. Under local anesthesia, through standardized clear corneal incisions, a continuous 5.7 mm curvilinear capsulorhexis was made, phacoemulsification and aspiration was performed. After lens removal, the posterior capsule was intact with a central localized opacity. A non-vascular fibrous stalk extending from the posterior lens capsule into the vitreous cavity was observed, with good mobility (Figure 3A). After filling the capsular bag with viscoelastics, a capsular tension ring (CTR) was implanted to support the capsular bag (Figure 3B). A transverse ellipse with a maximum diameter of 4 mm continuous curvilinear posterior capsulotomy was made, anterior vitrectomy was performed and the fibrous stalk was removed (Figure 3C). A posterior chamber monofocal IOL was then implanted into the capsular bag (Figure 3D). The incision was sutured watertight. No intraoperative complications such as vitreous hemorrhage, hyphema, elevated IOP, or retinal detachment occurred.

### Postoperative follow-up

**Figure 4:**
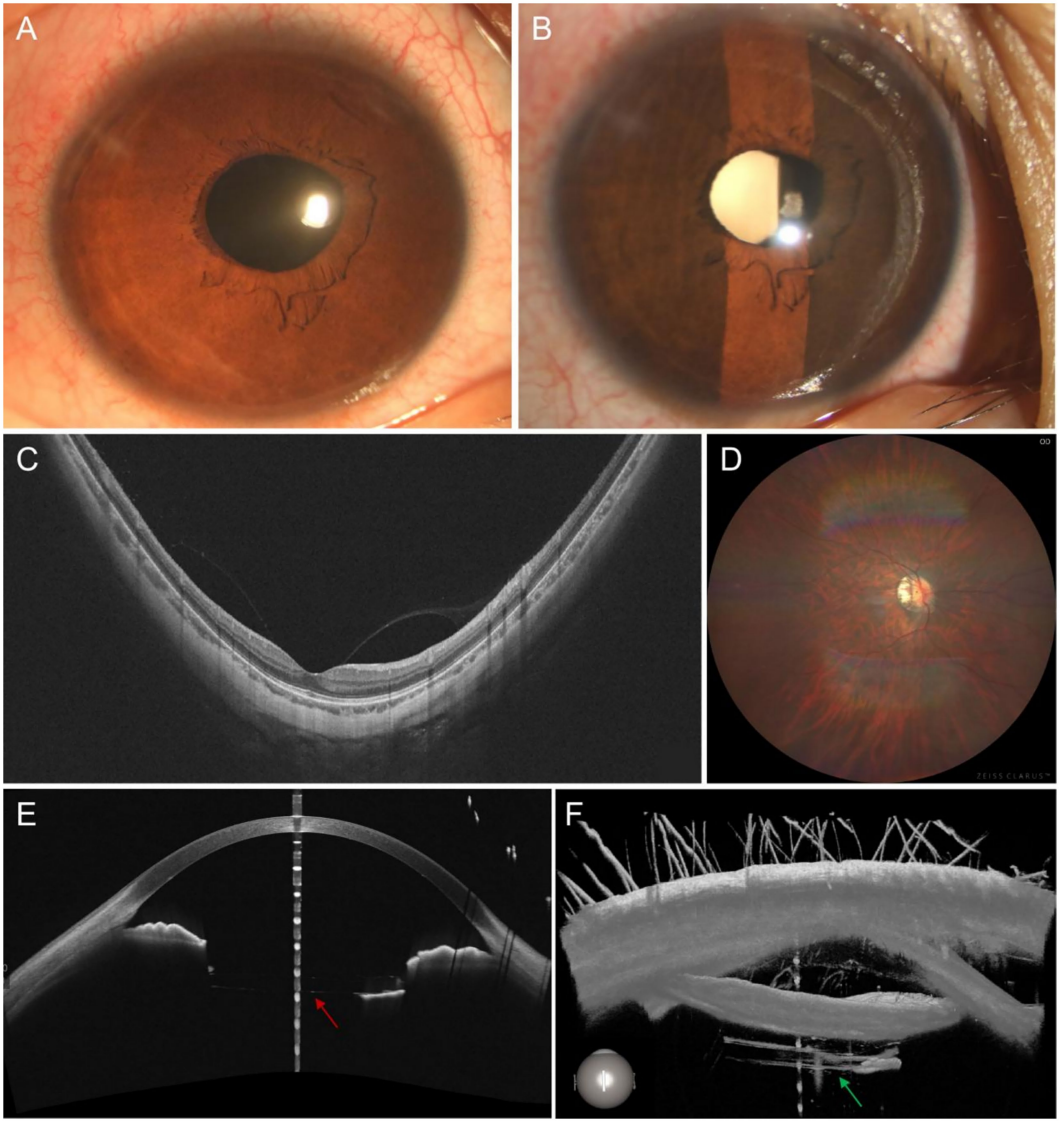
Post-operative examinations. **(A,B)** Anterior images one month after surgery; **(C)** post-op macular OCT revealed no abnormalities; **(D)** post-op fundus photo showed tigroid-patterned appearance and peripapillary atrophy; **(E)** AS-OCT nine months after surgery showed IOL in position (red arrow); **(F)** 3D AS-OCT nine months after surgery showed well-positioned IOL (green arrow).

The patient received post-op topical antibiotics and anti-inflammatory treatment. One month after surgery, the corneal suture was removed (Figures 4A,B). Two months after surgery, her BCVA was improved to 0.6. Postoperative examination showed clear cornea, normal anterior chamber depth and well-positioned IOL. Postoperative IOP were between 14 and 18 mmHg during follow-up visits. Macular OCT and fundus photography were performed, revealing no abnormalities in the macula or peripheral retina (Figures 4C,D). AS-OCT revealed that the preserved peripheral posterior capsule ensured an adequate capsular support for the IOL and the intraocular lens was well-centered in spite of the zonular defect (Figures 4E,F). At the nine-month postoperative follow-up, the visual acuity remained stable. The patient noted occasional linear floaters in the right eye but reported overall satisfaction with her postoperative visual outcome. Due to the patient’s long axial length, she was advised to avoid strenuous activities or ocular trauma, and attend follow-up visits every 6 months for dilated examinations to assess the IOL position and peripheral retinal status. The timeline of patient treatment and prognosis is presented in Table 1.

**Table 1:** Timeline of patient management.

Time	Event/Finding	Intervention	Outcome
Childhood	Poor vision in OD, myopia and amblyopia	Spectacles	Persistent poor vision
Age 0–13	Progressive, painless vision loss in OD	None	Gradual decline to 0.01
Age 23	Diagnosis of anterior PFV and lenticular coloboma	Phaco+CTR + posterior capsulotomy+ anterior vitrectomy+IOL	None
1 month post-op	BCVA 0.4	Corneal suture removal	BCVA improved
2 month post-op	BCVA 0.6	None	BCVA improved
9 month post-op	BCVA 0.6 occasional floaters	None	Stable, satisfied

## Discussion and conclusions

This case report describes a patient with anterior PFV associated with lenticular coloboma, presenting the following characteristics: (i) The patient had PFV combined with lenticular coloboma, which is rarely reported; (ii) AS-OCT detected the post-lenticular fibrous stalk while B-scan ultrasound revealed no vascular stalk or intraocular mass; (iii) The patient was treated with an optimized surgery of cataract and anterior vitrectomy, during which a capsular tension ring implantation was made and an IOL was implanted into capsular bag in one surgery.

PFV is a rare congenital disease with normally bad visual outcome. Patients with PFV may also have other ocular developmental abnormalities, such as leukokoria, microphthalmia, dysplastic optic nerve, retinal fold and tractional retinal detachment. Among these patients, the prognosis of purely anterior PFV is relatively favorable. Anterior PFV accounting for approximately 25 to 60% of cases, and is characterized by the proliferation of a retrolental fibrovascular membrane (2–4). This membrane progressively covers the posterior lens surface, invades the ciliary processes, and leads to their elongation and centripetal traction (2, 4, 5). Consequently, a spectrum of anterior segment abnormalities develops, including leukocoria, microphthalmia, cataract, shallow anterior chamber, secondary glaucoma, intralenticular hemorrhage, persistent pupillary membrane, iris vascular remnants, iris coloboma and ectropion uveae (3–5).

However, the association of PFV with lens anomalies is rarely reported. In 2018, Khokhar et al. reported a case of bilateral posterior lenticonus associated with PFV which indicate that hyaloid artery may cause traction of posterior capsule and lead to posterior lenticonus (9). In this case, the patients underwent lens aspiration and the IOL was implanted in the sulcus in both eyes. In 2018, Gonzalez et al. reported a 4 year-old patient with the concurrence of PFV, lenticular coloboma and a multiloculated ciliary body mass. This patient did not receive surgical treatment but had amblyopia treatment (10).

We believe this case to be the first instance of PFV associated with a lens coloboma reported in adult. The pathogenesis of PFV is characterized by the failure of the primary vitreous and hyaloid vasculature to regress normally, resulting in the formation of a retrolental fibrovascular membrane that drives subsequent developmental abnormalities. New evidence indicates that phagocytic cells, especially microglia and macrophages, are critical mediators of physiological ocular vascular regression during development, and their dysfunction may also contribute to the process in PFV (11). The possible developmental mechanism in this case is as follows: during the formation of the Cloquet’s canal, the regression of the primary vitreous coincides temporally and spatially with the development of the tertiary vitreous, which forms the zonular fibers. An incompletely regressed Cloquet’s canal forms a fibrous stalk connected to the optic disc, and its traction force causes the nasal equator of the lens to adhere to the ciliary processes. Due to the contact inhibition characteristics of epithelial cells, the lens surface in contact with the ciliary processes and provides a favorable substrate for the proliferation of pigment epithelium. As a result, a portion of the pigment epithelium extends posteriorly along the posterior lens capsule. The elongated ciliary processes obstruct the tertiary vitreous, preventing it from properly connecting to the normal lens equator. This leads to a defect in the nasal zonular fibers, subsequently resulting in a lens coloboma. As the eye continues to grow, the temporal zonular fibers develop normally, pulling the lens toward the temporal side. Simultaneously, with the gradual elongation of the axial length, the original Cloquet’s canal ruptures and becomes detached, floating freely behind the lens, ultimately forming anterior PFV. The coexistence of both PFV and lens coloboma made the treatment of this case more challenging.

Unlike typical PFV presentation, the patient did not present with congenital microphthalmia; instead, the affected eye exhibited axial elongation, which appears inconsistent with previous understanding. Microphthalmia in PFV is most common in the combined type, followed by the posterior type, while the anterior type has the lowest incidence and mildest degree (3). The pathogenesis of microphthalmia involves failed regression of the primary vitreous and hyaloid vasculature, along with fibrovascular membrane contraction that restricts ocular expansion, resulting in secondary growth arrest. However, in this case, only a fibrovascular stalk was present behind the lens, indicating weak tractional forces and reduces impact on ocular development. Postnatally, the refractive media opacity caused form deprivation amblyopia, which induced secondary axial elongation, leading to the paradoxical finding of a long axial length at adult presentation. Furthermore, a study by Xiang et al. on children aged 3–12 years found that posterior ciliary muscle thickness in myopic eyes was positively correlated with axial length, suggesting that ciliary body elongation may also be associated with abnormal axial development (12).

The diagnosis of PFV primarily relies on the typical fibrovascular stalks in the vitreous cavity. For patients with corneal or lens opacities that prevent direct observation of retrolental fibrous stalks, B-mode ultrasound often serves as the main auxiliary diagnostic method (7). Historically, OCT has played a limited role in the diagnosis of PFV. Hamza et al. reported a case where OCT assisted in diagnosing PFV by differentiating between optic disc traction and optic disc edema (13). The role of anterior segment OCT in the diagnosis of PFV has not yet been reported in the literature.

However, anterior segment OCT demonstrates significant advantages in the diagnosis of lens developmental anomalies. Chen et al. performed 360-degree panoramic imaging of the lens in patients with lens colobomas using a swept-source anterior segment optical coherence system, and for the first time observed continuous and blunt-edged margins in the areas of zonular deficiency (14).

In this case, AS-OCT clearly revealed the retrolental fibrovascular stalks which assisted in diagnosing anterior PFV, and provided a clear visualization of the lens and zonular defects. These findings indicate promising possibilities for the role of AS-OCT in in future ophthalmic diagnostics. In recent years, artificial intelligence has achieved remarkable success in the analysis of ophthalmic images, including fundus photography and optical coherence tomography. It has demonstrated substantial value in the differentiation of papilledema, the diagnostic classification of glaucoma, the staging assessment of retinal vascular and macular vascular diseases, myopic maculopathy, the diagnostic classification of cataracts and even endocrine and metabolic diseases (15–20). Artificial intelligence combined with AS-OCT technology holds broad prospects in future disease screening and diagnosis.

Vitrectomy combined with cataract surgery is the common treatment of PFV. For anterior PFV, to avoid potential adverse effects of pars plana vitrectomy (PPV) on the peripapillary retinal nerve fiber layer (pRNFL) and macular ganglion cell-inner plexiform layer (mGCIPL) (21), surgery combined with anterior vitrectomy is often the preferred treatment option. Through an analysis of 470 cases, Chen et al. proposed a new anatomical classification system for anterior PFV which can provide diagnostic indicators for occult PFV and offer guidance for surgical intervention (22). As for surgical strategies, anterior vitrectomy and fibrous stalk excision through a 4.0-mm posterior capsulotomy, and implantation of IOL into double capsulorhexis capsular bag is proved to be a safe and less invasive technology (6). Surgical treatment of lens coloboma is a great challenge due to the high risk of capsular fornix aspiration, zonular dialysis extension, vitreous herniation into the anterior chamber, intraocular lens decentration and closure of the capsular opening (23).

Ciliary sulcus implantation of IOL used to be a common choice. However, capsular tensor ring implantation combined with intracapsular IOL placement is a safer and more effective option (24, 25).

In this case, we combined and optimized these two approaches. A posterior capsulotomy was made after phacoemulsification of cataract through clear corneal incision, a capsular tensor ring was implanted to better support the capsule; fibrous stalk was removed and anterior vitrectomy was performed, and in the end, an IOL was implanted into the capsular bag. In this way, we employed the minimally invasive surgical approach and successfully completed the procedure in one single surgery for the patient.

In conclusion, this article reports a case of PFV associated with a lens coloboma discovered in adulthood, which expands our understanding of the PFV spectrum. AS-OCT played a significant role in the diagnosis of this condition, suggesting its potential for broader application in diagnosing anterior PFV in the future. In this case, the modified surgical technique, involving the implantation of a capsular tension ring, provided enhanced capsular bag stability. This approach ensured successful primary intraocular lens implantation into the capsular bag and offered greater assurance for long-term postoperative IOL stability.

## Data Availability

The original contributions presented in the study are included in the article/supplementary material, further inquiries can be directed to the corresponding authors.
